# Hepatitis B infection among pregnant and post-partum women living with HIV and on antiretroviral therapy in Kinshasa, DR Congo: A cross-sectional study

**DOI:** 10.1371/journal.pone.0216293

**Published:** 2019-05-09

**Authors:** Christian Mpody, Peyton Thompson, Martine Tabala, Noro Lantoniaina Rosa Ravelomanana, Fathy Malongo, Bienvenu Kawende, Frieda Behets, Emile Okitolonda, Marcel Yotebieng, for the CQI-PMTCT study team

**Affiliations:** 1 Division of Epidemiology, College of Public Health, The Ohio State University, Columbus, OH, United States of America; 2 Department of Pediatrics, University of North Carolina, Chapel Hill, NC, United States of America; 3 School of Public Health, University of Kinshasa, Kinshasa, Democratic Republic of Congo; 4 Department of Epidemiology, Gillings School of Global Public Health, University of North Carolina at Chapel Hill, Chapel Hill, NC, United States of America; University of Cincinnati College of Medicine, UNITED STATES

## Abstract

**Background:**

Hepatitis B virus (HBV) co-infection in HIV-infected individuals increases the risk of hepatic complications and mortality. Further, the risk of perinatal HBV transmission increases among HBV/HIV co-infected pregnant women. Although HBV is endemic in the Democratic Republic of Congo, there is little data on HBV/HIV co-infection. We aimed to assess the burden and risk factors of HBV surface antigen (HBsAg) positivity among HIV-infected pregnant and post-partum women.

**Methods:**

This cross-sectional study was conducted as part of an ongoing trial to assess the effect of data-driven continuous quality improvement interventions (CQI) for optimal prevention of mother-to-child transmission (PMTCT) of HIV (CQI-PMTCT study, NCT03048669). In each of the 35 health zones of Kinshasa province, all HIV-infected pregnant or breastfeeding women (≤1 year post-delivery) presenting for care in one of the three busiest maternal and child health clinics of the health zone were tested for HBsAg using Alere Determine, Japan. We used logistic regression with general estimating equation accounting for within-clinic clustering to assess risk factors of HBsAg positivity.

**Results:**

Between November 2016 and June 2018, a total of 1377 women, all on antiretroviral therapy, were tested for HBsAg. Overall, 4.7% [95% binomial confidence interval (CI): 3.7%-5.7%] tested positive for HBsAg. HBsAg prevalence was 3.3% (95% CI: 2.1%-4.8%) for women tested during pregnancy, 4.5% (2.5%-7.4%) for those tested at delivery, and 8.5% (5.6%-12.2%) for those tested post-partum (*P*_trend_ = 0.001). In multivariate models including socio-economic status (SES), type of care facility, duration of antiretroviral therapy, HIV viral load, and self-reported intimate partner violence (IPV), lowest tertile of SES, ≤ 6 months of ART, and IPV were all consistently and positively associated with higher prevalence of HBsAg across pregnancy, delivery, and postpartum period while been tested in a health centre or having a viral load ≥ 1000 copies/mL were consistently associated with lower prevalence. However, only the association with IPV (OR = 2.74, 95% CI: 1.10–6.84) and viral load between 40–1000 copies/ml (OR = 4.28, 95% CI: 1.22–15.01) achieved statistical significance among pregnant women.

**Conclusion:**

This study revealed an overall high prevalence of HBsAg among HIV-infected pregnant and post-partum women in Kinshasa with the latter showing the highest HBsAg prevalence. Among pregnant women, intimate partner violence was independently and statistically associated with HBsAg positivity, requiring further investigation.

## Introduction

In its 2016 Global Health Sector Strategy, the WHO called for reduction of viral hepatitis incidence and mortality by 90% and 65%, respectively [1]. Hepatitis B (HBV) virus, the most common cause of chronic hepatitis worldwide, is responsible for 275 million cases of chronic hepatitis, most of which are in the African and Western pacific regions [2]. HBV is endemic in the Democratic Republic of the Congo (DRC) [3–7] were the prevalence is higher among pregnant women than the general population: 5% versus 3%, respectively [5].

Co-infection with HIV and HBV, both of which can be transmitted vertically from mother to child or horizontally through exposure to infected blood, leads to more rapid escalation of hepatic complications [8, 9]. The risk of vertical transmission of HBV is greater among women living with HIV compared to those who are HIV-negative [10, 11]. Furthermore, HBV-infected infants born to HBV/HIV co-infected mothers are at higher risk of hepatic complications and death compared to those born to HBV mono-infected women [10].

Vertical transmission can be averted with antiviral treatment of pregnant women (for prevention of both HIV and HBV) and infant vaccination (for prevention of HBV). However, HBV testing of pregnant women is not part of routine care in the DRC. In order to implement appropriate mother-to-child prevention strategies among women living with HIV, it is important to first determine the prevalence of HIV-HBV co-infection.

Despite the endemicity of HBV in the DRC and the knowledge that its clinical course is worsened by co-infection with HIV, there is very little epidemiological data on HBV/HIV co-infection in DRC. The objective of this study was two-fold: first, to investigate the prevalence of HBV among HIV-positive pregnant and post-partum women; and, secondly, to assess the risk factors associated with HBV/HIV co-infection.

## Methods

### Study design and setting

This cross sectional study was conducted as part of an ongoing randomized trial to investigate the effect of data-driven continuous quality interventions (CQI) on long term ART outcomes among pregnant and breastfeeding women receiving care in the Kinshasa province (CQI-PMTCT study, NCT03048669) [12]. In each of the 35 health zones in the province, the three busiest maternal and child health (MCH) facilities were selected to be part of the study.

The parent CQI-PMTCT study is prospective in nature, enrolling women at pregnancy, immediately (1–3 days) after delivery, and in the first 12 months of the post-partum period. At enrollment, HBsAg status of eligible participant was assessed. The study was approved by the Ohio State University Institutional Review Board and the Kinshasa School of Public Health Ethical Committee.

### Inclusion and exclusion criteria

All HIV-infected pregnant or breastfeeding women receiving care at any of the selected MCH facilities between November 2016 and June 2018 were eligible for the parent study. Women were excluded if they refused to participate or if their breastfeeding infant was more than 12 months old or were not on triple antiretroviral therapy (ART). Because of the initial delay in obtaining test kits, participants who were enrolled before testing for HBsAg started were excluded for the present analysis.

### Data collection

Eligible participants were referred to study staff to which they provided informed consent before enrollment in the study. Such referral occurred upon presentation for routine visits any time during pregnancy, immediately (1–3 days) after delivery, or during the well-child clinic visits in the postpartum period. For those who consented to be part of the study, a structured questionnaire was used to collect demographic and clinical information. Study staff also collected data on health facility characteristics using a structured questionnaire.

### Outcome variables

The outcome for this study was the presence or absence of HBsAg in a participant’s blood, which was assessed by using the Alere Determine rapid diagnostic test. HBsAg test was conducted according to the manufacturer instructions. Following the structured interview, a capillary blood sample was obtained via finger prick. A 50μL of blood was added to the test sample pad, immediately after the blood was absorbed, and a drop of chase buffer was also applied to the sample pad. The preparation was left on a flat surface for a minimum of 15 minutes. Test result was read according to manufacturer instructions as positive, negative, or indeterminate (if the control bar was not visible). Women who refused to be tested for HBsAg could still participate in the main study, and their socio-demographic characteristics did not differ from that of women who had HBsAg results. Participants’ data were recorded in structured forms and taken to the study office where they were entered in an electronic database.

### Covariates

Other variables considered in this analysis included participants’ socio-demographic and clinical characteristics as well as selected health facility characteristics. Participants’ socio-demographic characteristics included maternal age (≤ 24, 25–34, or 35+), marital status (married/cohabitating vs. Divorced/separated/ widow/ never married), educational level (primary or less, secondary, or tertiary), alcohol consumption (no vs. yes), and socioeconomic status (SES) measured by a wealth index. We calculated the wealth index score by performing a principal components analysis of factors including years of education, average number of household members per room, number of sleeping beds in the household, type of household water source (communal vs. private pipe), cooking fuel type (electrical stove vs. wood/charcoal), and ownership status (yes vs no) for several household’s goods (mobile phone, radio, fridge, vehicle, bike, and motorcycle). The first component explained 20.7% of variability and was categorized into tertiles to obtain the wealth index: 0 (lowest SES), 1, and 2 (highest SES).

Clinical characteristics included timing of HBsAg testing (during pregnancy, at delivery, and during the post-partum period), duration of ART in months (≤6, 6–24, or >24), disclosure of HIV status to anyone (yes vs. no), primigravida (no vs. yes), report of any intimate partner violence (IPV; yes vs. no), HIV viral load (< 40, 40–1000, and > 1000 copies/mL), and ART regimen (tenofovir/lamivudine/efavirenz, zidovudine/lamivudine/nevirapine, or other). Facility characteristics included location (urban vs. peri-urban/rural) and type of facility (hospital vs. health center).

### Statistical analysis

We calculated the proportion of participants with a positive HBsAg test as a measure of hepatitis B virus prevalence and their 95% binomial confidence interval (CI). Bivariate and multivariable logistic regressions were used to estimate prevalence odds ratios (OR) and 95% CI comparing the prevalence of HBsAg across levels of covariates. Generalized estimating equation was used to account for potential clustering at the level of health facilities. Variables that were found to be statistically associated, at the alpha level of 0.20, with HBsAg positivity were included in the multivariable model [13]. After assessment and resolution of collinearity, all remaining variables were retained in the final model. All analyses were conducted using SAS, version 9.3 (SAS Institute Inc., Cary, North Carolina), and STATA/IC version 14.0 (StataCorp LP, College Station, Texas) and all tests unless otherwise indicated, were conducted using a two-sided 0.05 significance level, without correction for multiple comparisons.

## Results

**Fig 1:**
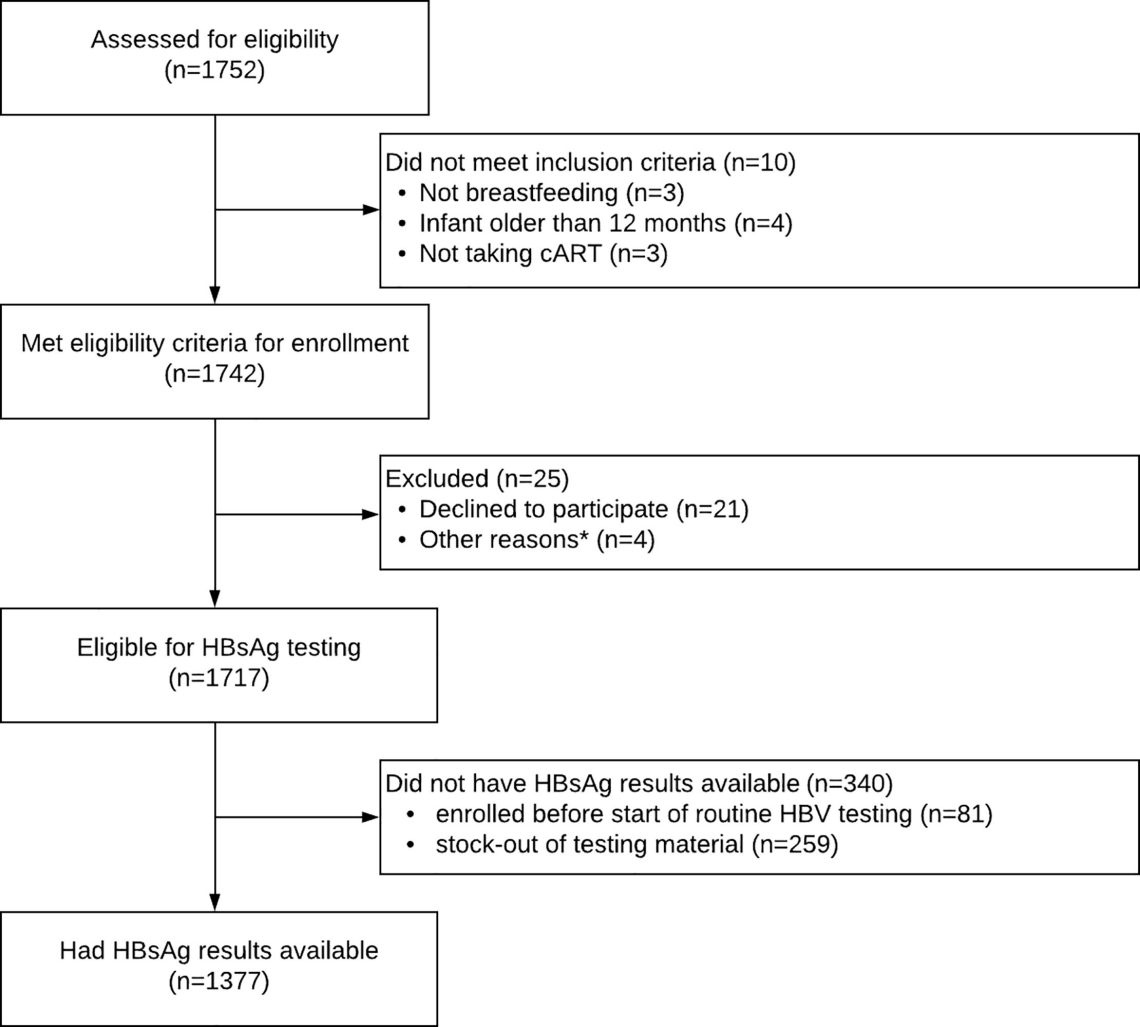
Participants’ flowchart. *Other reasons include hearing-impairment for one participant and intent to transfer to a different clinic for three participants.

Between November 2016 and June 2018, the parent study enrolled 1717 women out of 1752 who were initially assessed for eligibility. Of these, 81 (4.7%) were enrolled before the routine HBV testing started, 259 (15.1%) were not tested due to stock-out of prick test device, and 21 (1.2%) women declined to provide blood sample; leaving 1377 eligible for analysis (Fig 1). Of the 1377 women with available HBsAg results who were included in this analysis, 55.1% (759) were tested during pregnancy, 22.6% (311) were tested at delivery, and 22.3% (307) were tested during the post-partum period (Table 1). Overall, most women (93.5%) were enrolled in urban areas and 56.1% were enrolled in hospital settings while 43.9% were recruited at health centres. The median age was 32 years [interquartile range (IQR): 27–36]. The majority (90.8%) of women were multigravida. About two-third (66.8%) of women were married/cohabitating, 71.2% reported not drinking alcohol, 70.0% reported some secondary education, and 59.6% reported emotional or physical or sexual partner violence within the last 12 months. Except for 16 (1.3%) women, all participants were on lamivudine containing regimen, and 84.6% were on Tenofovir and Lamivudine containing regimen. Just over half (52.2%) of women had disclosed their HIV status to someone; 52.5% and 8.6% had viral load <40 copies/ml and between 40–1000 copies/ml; 37.5% had been on ART for ≤6 months and 15.7% had been on ART for 6–24 months (Table 1). Women who did not have HBsAg results did not differ from those who were retained in the analysis, except for location of facility and timing of enrollment. Those who had HBsAg results were less likely to be in care in a peri-urban/rural facility (6.5% vs 11.2%, *P-*value = 0.003), and more likely to be enrolled during the post-partum (29.1% vs 22.3%, *P-*value = 0.016) (S1 Table).

**Table 1:** Socio-demographic and clinical characteristics of 1377 women living with HIV stratified by timing of HBsAg testing*.

Characteristics	Overall		Pregnant women		Women at delivery		Post-partum women	
	N = 1377†		759†	(55.1)	311†	(22.6)	307†	(22.3)
	No	%‡	No	%‡	No	%‡	No	%‡
**Location of facility§** **attended**								
Urban	1288	(93.5)	710	(93.5)	295	(94.9)	283	(92.2)
Peri-urban/Rural	89	(6.5)	49	(6.5)	16	(5.1)	24	(7.8)
**Type of facility§** **of care**								
Hospitals	772	(56.1)	414	(54.5)	190	(61.1)	168	(54.7)
Health centres	605	(43.9)	345	(45.5)	121	(38.9)	139	(45.3)
**Age (median [IQR])**	**32**	**[27,36]**	**32**	**[27,36]**	**32**	**[27,36]**	**32**	**[27,36]**
35+	438	(32.4)	230	(30.3)	105	(35.0)	103	(35.0)
25–34	716	(53.0)	422	(55.7)	147	(49.0)	147	(50.0)
≤ 24	198	(14.6)	106	(14.0)	48	(16.0)	44	(15.0)
**Marital status**								
Married/cohabitating	902	(66.8)	516	(68.3)	200	(66.7)	186	(63.3)
Divorced/separated/ widow/never married	448	(33.2)	240	(31.7)	100	(33.3)	108	(36.7)
**Alcohol consumption**								
No	963	(71.2)	527	(69.5)	208	(69.3)	228	(77.6)
Yes	389	(28.8)	231	(30.5)	92	(30.7)	66	(22.4)
**Educational level**								
Tertiary	216	(16.0)	141	(18.6)	41	(13.7)	34	(11.6)
Secondary	946	(70.0)	520	(68.6)	211	(70.6)	215	(73.1)
Primary	189	(14.0)	97	(12.8)	47	(15.7)	45	(15.3)
**SES in quintile¶**								
3 (Highest)	400	(32.8)	270	(36.4)	72	(29.4)	58	(24.9)
2	407	(33.4)	246	(33.2)	95	(38.8)	66	(28.3)
1(Lowest)	412	(33.8)	225	(30.4)	78	(31.8)	109	(46.8)
**Primigravida**								
Yes	125	(9.2)	77	(10.1)	29	(9.7)	19	(6.5)
No	1228	(90.8)	682	(89.9)	271	(90.3)	275	(93.5)
**Any intimate partner violence#**								
No	805	(59.6)	459	(60.7)	178	(59.3)	168	(57.1)
Yes	545	(40.4)	297	(39.3)	122	(40.7)	126	(42.9)
**HIV RNA viral load**								
> 1000 copies/mL	503	(38.9)	270	(37.7)	133	(46.0)	100	(34.7)
40–1000 copies/mL	111	(8.6)	68	(9.5)	22	(7.6)	21	(7.3)
< 40 copies/mL	679	(52.5)	378	(52.8)	134	(46.4)	167	(58.0)
**ART Regimen**								
TDF+3TC+FEV	1029	(84.6)	570	(83.7)	244	(89.7)	215	(81.8)
AZT+3TC+NVP	171	(14.1)	103	(15.1)	25	(9.2)	43	(16.4)
Other	16	(1.3)	8	(1.2)	3	(1.1)	5	(1.9)
**Duration of ART**								
> 24 months	628	(46.8)	344	(45.6)	131	(44.1)	153	(52.4)
7–24 months	211	(15.7)	86	(11.4)	35	(11.8)	90	(30.8)
≤ 6 months	504	(37.5)	324	(43.0)	131	(44.1)	49	(16.8)
Disclosure of HIV status**								
Yes	715	(52.2)	403	(53.2)	145	(46.6)	167	(55.3)
No	656	(47.8)	355	(46.8)	166	(53.4)	135	(44.7)

*The analytical sample was derived from the enrollment data of an ongoing cluster randomized controlled trial, aimed at evaluating the effect of data-driven continuous quality improvement on long-term ART outcomes in Kinshasa, Democratic Republic of Congo. We retained participants that had available data on HBsAg testing.

†Frequencies might not add up to n, because of missing data.

‡ Column percentage.

§Facility at which participant was enrolled/tested.

¶Calculated using principal component analysis and categorized in three groups: the lower first two tertiles, the middle tertile, and the last two tertile.

#Self-report of emotional or physical or sexual partner violence.

**Self-report of disclosure of HIV status to anyone. Abbreviations: No, Number; VL, Viral load; SES, Socio-economic status; RNA, Ribonucleic acid, ART, Antiretroviral therapy.

Of the 1377 women with HBsAg results, 65 tested positive, resulting in an overall seroprevalence of 4.7% (95% CI: 3.7%-5.7%). The prevalence of HBsAg positivity was greatest among women who were tested during the post-partum period, followed by women tested at delivery, and finally by women tested during pregnancy (8.5% vs 4.5% vs 3.3%, trend *P-*value = 0.001). The odd ratios of HBsAg positivity among women who tested at delivery and postpartum period, relative to those who tested during pregnancy were 1.38 (95%CI:0.71, 2.69) and 2.71 (95%CI:1.54, 4.77) respectively (Table 2)

**Table 2:** Prevalence of HBsAg by timing of testing among 1377 women living with HIV in Kinshasa.

Timing of testing	n(%)†	Frequency HBsAg +	Prevalence in %‡	OR(95% CI)§	Trend test *P*-value
During pregnancy	759(55.1)	25	3.3	Ref	0.001
At delivery	311(22.6)	14	4.5	1.38 (0.71, 2.69)	
During post-partum	307(22.3)	26	8.5	2.71 (1.54,4.77)	
**Overall**	**1377**	**65**	**4.7**	**—**	—

*The analytical sample was derived from the enrollment data of an ongoing cluster randomized controlled trial, aimed at evaluating the effect of data-driven continuous quality improvement on long-term ART outcomes in Kinshasa, Democratic Republic of Congo. We retained participants that had available data on HBsAg testing.

†Column percentages. Frequencies might not add up to n, because of missing data.

‡Row percentages.

§Estimated by crude logistic regression. Abbreviations: CI: confidence interval; OR, Odds ratio.

Several associations emerged when we performed sub-analyses of results based on timing of HBsAg testing (Table 3). Among pregnant women, reporting of intimate partner violence within the last 12 months was associated with relative higher odds of HBsAg positivity (OR:2.81, 95%CI:1.23,6.41). Compared to pregnant women with viral load > 1000 copies/ml, those with viral load between 40–1000 copies/ml had relative higher odds of HBsAg positivity (OR:3.44, 95%CI:1.01,11.66). Among women tested at delivery, having viral load between 40–1000 copies/ml was associated with relative higher odds of HBsAg positivity (OR:10.70, 95%CI:1.67, 68.75). Among women tested during the post-partum period, the prevalence of HBsAg was lower for those who received care in health centers relative to hospital settings (5% vs 11.3%; OR = 0.40, 95%CI:0.18–0.92) and higher for those who reported alcohol consumption (15.2% vs 6.6%; OR = 2.54, 95%CI:1.09, 5.94).

**Table 3:** Bivariate associations between health facility, socio-demographic and clinical characteristics and HBsAg status among 1377 women living with HIV stratified by timing of testing.

Characteristics	During pregnancy (n = 759)					At delivery (n = 311)					During Post-partum (n = 307)				
	n†	HBV +	%‡	cOR	95% CI	n†	HBV +	%‡	cOR	95% CI	n†	HBV +	%‡	cOR	95% CI
**Location of facility§ attended**															
Urban	710	22	(3.1)			295	13	(4.4)			283	25	(8.8)		
Peri-urban/Rural	49	3	(6.1)	2.01	(0.57,7.09)	16	1	(6.3)	1.46	(0.18,11.68)	24	1	(4.2)	0.47	(0.07,3.32)
**Type of facility§ of care**															
Hospital	414	17	(4.1)			190	9	(4.7)			168	19	(11.3)		
Health center	345	8	(2.3)	0.55	(0.23,1.32)	121	5	(4.1)	0.89	(0.30,2.62)	139	7	(5.0)	0.40	(0.18,0.92)
**Age**															
35+	230	8	(3.5)			105	3	(2.9)			103	12	(11.7)		
25–34	422	14	(3.3)	0.93	(0.39,2.24)	147	9	(6.1)	2.17	(0.59,8.05)	147	9	(6.1)	0.52	(0.21,1.27)
≤ 24	106	3	(2.8)	0.80	(0.21,3.04)	48	2	(4.2)	1.35	(0.22,8.43)	44	4	(9.1)	0.79	(0.24,2.59)
**Marital status**															
Married/cohabitating	516	16	(3.1)			200	9	(4.5)			186	15	(8.1)		
Divorced/separated/ widow/never married	240	7	(2.9)	0.93	(0.38,2.28)	100	5	(5)	1.15	(0.38,3.50)	108	10	(9.3)	1.15	(0.50,2.64)
**Alcohol consumption**															
No	527	21	(4)			208	11	(5.3)			228	15	(6.6)		
Yes	231	4	(1.7)	0.40	(0.13,1.22)	92	3	(3.3)	0.63	(0.18,2.21)	66	10	(15.2)	2.54	(1.09,5.94)
**Educational level**															
Tertiary	141	5	(3.5)			41	1	(2.4)			34	2	(5.9)	.	
Secondary	520	16	(3.1)	0.86	(0.31,2.37)	211	8	(3.8)	1.63	(0.19,13.68)	215	19	(8.8)	1.43	(0.33,6.18)
Primary	97	4	(4.1)	1.15	(0.30,4.38)	47	4	(8.5)	3.85	(0.40,36.75)	45	4	(8.9)	1.46	(0.26,8.13)
**SES in tertile¶**															
3 (Highest)	270	4	(1.5)			72	3	(4.2)			58	5	(8.6)		
2	246	10	(4.1)	2.81	(0.87,9.07)	95	5	(5.3)	1.30	(0.30,5.60)	66	5	(7.6)	0.89	(0.25,3.13)
1(Lowest)	225	10	(4.4)	3.08	(0.95,9.95)	78	4	(5.1)	1.24	(0.27,5.72)	109	11	(10.1)	1.07	(0.36,3.21)
**Primigravida**															
Yes	77	1	(1.3)			29	2	(6.9)			19	3	(15.8)		
No	682	24	(3.5)	2.76	(0.38,20.25)	271	12	(4.4)	0.63	(0.13,3.00)	275	22	(8)	0.44	(0.12,1.59)
**Any intimate partner violence#**															
No	459	9	(2)			178	8	(4.5)			168	12	(7.1)		
Yes	297	16	(5.4)	2.81	(1.23,6.41)	122	6	(4.9)	1.10	(0.37,3.24)	126	13	(10.3)	1.47	(0.65,3.31)
**ART Regimen**															
TDF+3TC+FEV	570	20	(3.5)			244	12	(4.9)			215	17	(7.9)		
AZT+3TC+NVP	103	2	(1.9)	0.55	(0.13,2.37)	25	0	(0.0)	-	-	43	7	(16.3)	2.31	(0.89,5.95)
Other	8	0	(0.0)	-	-	3	0	(0.0)	-	-	5	0	(0)	-	-
**HIV RNA viral load**															
> 1000 copies/mL	270	6	(2.2)			133	2	(1.5)			100	8	(8)		
40–1000 copies/mL	68	5	(7.4)	3.44	(1.01,11.66)	22	3	(13.6)	10.70	(1.67,68.75)	21	3	(14.3)	1.95	(0.47,8.10)
< 40 copies/mL	378	11	(2.9)	1.32	(0.48,3.59)	134	8	(6.0)	4.30	(0.88,21.06)	167	14	(8.4)	1.06	(0.43,2.62)
**Duration of ART**															
> 24 months	344	9	(2.6)			131	4	(3.1)			153	11	(7.2)		
7–24 months	86	1	(1.2)	0.45	(0.06,3.44)	35	3	(8.6)	2.97	(0.63,14.00)	90	8	(8.9)	1.29	(0.50,3.32)
≤ 6 months	324	15	(4.6)	1.80	(0.78,4.15)	131	7	(5.3)	1.80	(0.51,6.30)	49	5	(10.2)	1.47	(0.48,4.45)
Disclosure of HIV status**															
Yes	403	13	(3.2)			145	6	(4.1)			167	17	(10.2)		
No	355	12	(3.4)	1.04	0.472.30	166	8	(4.8)	1.18	(0.40,3.51)	135	9	(6.7)	0.63	(0.27,1.46)

*The analytical sample was derived from the enrollment data of an ongoing cluster randomized controlled trial, aimed at evaluating the effect of data-driven continuous quality improvement on long-term ART outcomes in Kinshasa, Democratic Republic of Congo. We retained participants that had available data on HBsAg testing.

†Frequencies might not add up to n, because of missing data.

‡Percentage of row.

§Facility at which participants attend PMTCT visits.

¶Calculated using principal component analysis and categorized in three groups: the lower first two quintiles, the middle quintile, and the last two quintiles.

#Self-report of emotional or physical or sexual partner violence during past 12 months.

**Self-report of disclosure of HIV status to anyone. Abbreviations: No, Number; VL, Viral load; SES, Socio-economic status; RNA, Ribonucleic acid, ART, Antiretroviral therapy.

In the multivariable analyses, lower SES, lower duration of ART, viral load between 40–1000 copies/ml and reporting of IPV in the past 12 months were all associated with relative higher odds of HBsAg positivity particularly for women tested during pregnancy for whom reporting any IPV in the past 12 months was statistically significantly (OR:2.74, 95%CI:1.10,6.84) [Table 4]. On the other hand, participants who were tested in a health center as opposed to a hospital had relative lower odds of testing positive, although not statistically significant.

**Table 4:** Multivariable associations between health facility, socio-demographic and clinical characteristics and HBsAg status among 1377 women living with HIV stratified by timing of testing*.

Characteristics	Pregnant women (n = 759)		Women at delivery (n = 311)		Post-partum women (n = 307)	
	aOR†	95% CI	aOR†	95% CI	aOR†	95% CI
**Type of facility‡ of care**						
Hospital						
Health centre	0.50	(0.19,1.30)	0.64	(0.16,2.60)	0.55	(0.19,1.60)
**SES in tertile§**						
3 (Highest)						
2	3.31	(0.98,11.19)	1.09	(0.21,5.66)	1.02	(0.23,4.54)
1(Lowest)	3.15	(0.91,10.94)	1.30	(0.24,7.03)	2.47	(0.63,9.58)
**Any intimate partner violence¶**						
No						
Yes	2.74	(1.10,6.84)	1.99	(0.52,7.59)	1.78	(0.67,4.73)
**HIV RNA viral load#**						
> 1000 copies/mL						
40–1000 copies/mL	4.28	(1.22,15.01)	13.38	(1.90,94.18)	3.52	(0.70,17.59)
< 40 copies/mL	1.36	(0.48,3.88)	2.66	(0.50,14.22)	0.97	(0.32,2.89)
**Duration of ART**						
> 24 months						
7–24 months	0.46	(0.06,3.79)	3.91	(0.70,21.88)	1.76	(0.57,5.40)
≤ 6 months	1.92	(0.75,4.90)	2.05	(0.45,9.36)	2.27	(0.58,8.81)

*The analytical sample was derived from the enrollment data of an ongoing cluster randomized controlled trial, aimed at evaluating the effect of data-driven continuous quality improvement on long-term ART outcomes in Kinshasa, Democratic Republic of Congo. We retained participants that had available data on HBsAg testing.

†Obtained by logistic regression model, adjusted for all covariates in the table, and where general estimating equation was used to adjust for within health facilities clustering.

‡Facility at which participants attend PMTCT visits.

§Calculated using principal component analysis and categorized in three groups: the lower first two quintiles, the middle quintile, and the last two quintiles.

¶Self-report of emotional or physical or sexual partner violence in the past 12 months. Abbreviations: VL, Viral load; SES, Socio-economic status; RNA, Ribonucleic acid, ART, Antiretroviral therapy.

## Discussion

This study found an HBV prevalence of 4.7% among pregnant and post-partum women living with HIV in the Kinshasa Province, which is similar to the 5% estimates among pregnant women irrespective of HIV status reported in a recent pooled analysis of published studies in DRC [14]. Our estimated prevalence is also similar to those from other central and southern African countries, but lower than those seen in western Africa [15]. The mechanism behind this regional variation of HBV prevalence is unclear, but may in part be explained by differences in socio-cultural factors [16] and/or differences in viral genotype, as there is a known “East-West divide” of hepatitis B genotypes A & E in eastern versus western Africa, respectively [17, 18].

Vertical transmission of HBV, either in utero or peripartum is responsible for up to 50% of HBV infection worldwide [19]. The risk of vertical transmission can be reduced by 90% when vaccine coupled with HBV Immune Globulin are administered to the infant at birth [20, 21]. The remaining 10% accounts for vertical transmission occurring in pregnant women with high-level HBV DNA viremia and/or e antigen (HBeAg) positivity. Tenofovir and/or lamivudine administered to these high-risk women reduce HBV viral load and the risk of transmission [22–24]. HBV vaccine has been administered as part of DRC’s Expanded Program on Immunization since 2009 with relatively high coverage (85% of children in Kinshasa are fully immunized) [25]. In addition, because of their HIV status, virtually all participants in this study were on Tenofovir/lamivudine (85%) and 14% on lamivudine containing ART regimen.

As expected, women on ART for less than 6 months had twice the odds of HBsAg positivity than those on ART longer than 24 months. This finding was expected because virtually all participants were either on tenofovir or lamivudine containing ART regimens for HIV, antivirals that are also active against HBV [26, 27]. However, the finding that women with viral load between the limit of undetectable (40 copies/ml) and 1000 copies/ml had higher odds of HBsAg is not easy to explain. Because the association was not significant in postpartum women, we speculate that this high positivity might be due to recently diagnosed women, who are responsive to ART treatment, but are yet to be virologically suppressed. In addition, the apparent lower prevalence of HBsAg in women with viral load >1000 copies/ml might be due to women who have been on ART for longer duration and may have thus cleared HBsAg.

HBsAg prevalence was highest among women tested in the post-partum period. This may be explained by a reactivation of HBV infection following ART discontinuation during the post-partum period. While the prevalence of ART discontinuation during the post-partum period has not been well documented in DRC, emerging studies are assessing its impact on vertical transmission of HIV and/or prognosis of HIV infection[28]. However, a longitudinal design may be better suited to determine whether ART discontinuation in HBV/HIV co-infected patients is associated with higher prevalence of HBsAg. Regardless of the mechanism, our findings underline the need to investigate the implications of ART discontinuation, given that it may also increase HBV viral load, and thus the risk of HBV peripartum transmission [27]. The higher HBV prevalence in the post-partum period may alternatively be related to selection bias as substantial proportion of HIV-infected mothers in Kinshasa are lost to follow-up prior to six weeks postpartum [29]. If retention in care is associated either directly or indirectly with HBsAg positivity, this will result in high prevalence in our sample of retained women.

Finally, intimate partner violence was found to be the only predictor that remained significantly associated with HBV infection in the multivariable analysis. Women enrolled during pregnancy, who reported IPV within the past 12 months, had twice the odds of HBsAg positivity than those who did not report IPV. HBV is sexually transmitted and about one in five pregnant women living with HIV in Kinshasa report sexual abuses [30]. In addition, the trauma and stress associated with IPV may reduce adherence to ART [31, 32], which in turn may lead to HBV reactivation. IPV may also occur within a context of substance abuse among women [33], which has the potential to increase the risk of HBV. Future studies should focus on better defining the relationship between various forms of IPV and HBV among HIV-infected and uninfected women.

One of the main limitations of this study was its cross-sectional design. Thus, we were not able to infer causal association between ART compliance over time and higher HBsAg positivity. In addition, we were not able to follow participants and perform other testing, such as HBV viral load, to correlate this with ART discontinuation. Another limitation was the unavailability of HBsAg results among 21% of participants in the parent study. However, it was reassuring to observe that our analytical sample was comparable with the excluded women with regards to socio-demographic characteristics. Finally, the lack of additional HBV testing over time precludes us from being able to know whether HBV-positive women were acutely infected (in which case they would achieve clearance of the virus over time) or chronically infected (in which case they would risk development of hepatic complications).

## Conclusion

This study revealed a high prevalence of HBV among HIV-infected pregnant and post-partum women in Kinshasa, DRC. More research and funding should be directed towards the prevention of HBV in this population and stop further transmission. Women tested postpartum had the highest HBV prevalence (8.5%), requiring further investigation as it may indicate a higher risk of vertical transmission of both HIV and HBV.

## Supporting information

S1 Table Comparison of socio-demographic and clinical characteristics between women who were included in (HBsAg results available) and those excluded from the analytical sample (HBsAg results not available). Abbreviations: No, Number; VL, Viral load; SES, Socio-economic status; RNA, Ribonucleic acid, ART, Antiretroviral therapy.
(DOCX)
