# Supplementary material for: Hepatitis B infection among pregnant and post-partum women living with HIV and on antiretroviral therapy in Kinshasa, DR Congo: A cross-sectional study
Source: PLoS One. 2019 May 9;14(5):e0216293. doi: 10.1371/journal.pone.0216293 (PMC6508921; doi:10.1371/journal.pone.0216293)
Supplement: S1 Table — Abbreviations: No, Number; VL, Viral load; SES, Socio-economic status; RNA, Ribonucleic acid, ART, Antiretroviral therapy. (DOCX) [file pone.0216293.s001.docx]

| **S1 Table. Comparison of socio-demographic and clinical characteristics between women who were included in (HBsAg results available) and those excluded from the analytical sample (HBsAg results not available)*** | | | | | | | | | | |
| --- | --- | --- | --- | --- | --- | --- | --- | --- | --- | --- |
| **Characteristics** | Women eligible for HBsAg testing | |  | Women without HBsAg results | |  | Women with HBsAg results | |  | Chi-square *P*-value |
|  | N=1717† | |  | 340† | (19.8) |  | 1377† | (80.2) |  |  |
|  | No | %‡ |  | No | %‡ |  | No | %‡ |  |  |
| **Location of facility§ attended** |  |  |  |  |  |  |  |  |  |  |
| Urban | 1588 | (92.6) |  | 300 | (88.8) |  | 1288 | (93.5) |  | 0.003 |
| Peri-urban/Rural | 127 | (7.4) |  | 38 | (11.2) |  | 89 | (6.5) |  |  |
| **Type of facility§ of care** |  |  |  |  |  |  |  |  |  |  |
| Health centre | 743 | (43.3) |  | 138 | (40.8) |  | 605 | (43.9) |  | 0.302 |
| Hospital | 972 | (56.7) |  | 200 | (59.2) |  | 772 | (56.1) |  |  |
| **Age** |  |  |  |  |  |  |  |  |  |  |
| 35+ | 533 | (31.7) |  | 95 | (29.1) |  | 438 | (32.4) |  | 0.504 |
| 25-34 | 897 | (53.4) |  | 181 | (55.4) |  | 716 | (53.0) |  |  |
| ≤24 | 249 | (14.8) |  | 51 | (15.6) |  | 198 | (14.6) |  |  |
| **Timing of HBV testing** |  |  |  |  |  |  |  |  |  |  |
| During pregnancy | 921 | (53.6) |  | 162 | (47.6) |  | 759 | (55.1) |  | 0.016 |
| At delivery | 390 | (22.7) |  | 79 | (23.2) |  | 311 | (22.6) |  |  |
| During post-partum | 406 | (23.6) |  | 99 | (29.1) |  | 307 | (22.3) |  |  |
| **Marital status** |  |  |  |  |  |  |  |  |  |  |
| Married/cohabitating | 1138 | (67.9) |  | 236 | (72.2) |  | 902 | (66.8) |  | 0.063 |
| Divorced/separated/ widow/never married | 539 | (32.1) |  | 91 | (27.8) |  | 448 | (33.2) |  |  |
| **Alcohol consumption** |  |  |  |  |  |  |  |  |  |  |
| No | 1198 | (71.4) |  | 235 | (71.9) |  | 963 | (71.2) |  | 0.819 |
| Yes | 481 | (28.6) |  | 92 | (28.1) |  | 389 | (28.8) |  |  |
| **Educational level** |  |  |  |  |  |  |  |  |  |  |
| Tertiary | 263 | (15.7) |  | 47 | (14.4) |  | 216 | (16) |  | 0.483 |
| Secondary | 1186 | (70.7) |  | 240 | (73.4) |  | 946 | (70) |  |  |
| Primary | 229 | (13.6) |  | 40 | (12.2) |  | 189 | (14) |  |  |
| **SES in tertile¶** |  |  |  |  |  |  |  |  |  |  |
| 3 (Highest) | 504 | (33.5) |  | 104 | (36.6) |  | 400 | (32.8) |  | 0.472 |
| 2 | 496 | (33.0) |  | 89 | (31.3) |  | 407 | (33.4) |  |  |
| 1(Lowest) | 503 | (33.5) |  | 91 | (32.0) |  | 412 | (33.8) |  |  |
| **Primigravida** |  |  |  |  |  |  |  |  |  |  |
| Yes | 154 | (9.2) |  | 29 | (8.9) |  | 125 | (9.2) |  | 0.835 |
| No | 1526 | (90.8) |  | 298 | (91.1) |  | 1228 | (90.8) |  |  |
| **Any intimate partner violence#** |  |  |  |  |  |  |  |  |  |  |
| No | 997 | (59.5) |  | 192 | (58.7) |  | 805 | (59.6) |  | 0.763 |
| Yes | 680 | (40.5) |  | 135 | (41.3) |  | 545 | (40.4) |  |  |
| **HIV RNA viral load** |  |  |  |  |  |  |  |  |  |  |
| VL > 1000 copies/mL | 978 | (61.5) |  | 188 | (63.5) |  | 790 | (61.1) |  | 0.441 |
| VL ≤ 1000 copies/mL | 611 | (38.5) |  | 108 | (36.5) |  | 503 | (38.9) |  |  |
| **Duration of ART** |  |  |  |  |  |  |  |  |  |  |
| ≤ 6 months | 767 | (46.1) |  | 139 | (43.2) |  | 628 | (46.8) |  | 0.167 |
| 7-24 months | 255 | (15.3) |  | 44 | (13.7) |  | 211 | (15.7) |  |  |
| > 24 months | 643 | (38.6) |  | 139 | (43.2) |  | 504 | (37.5) |  |  |
| **Disclosure of HIV status**** |  |  |  |  |  |  |  |  |  |  |
| Yes | 872 | (51.2) |  | 157 | (47.4) |  | 715 | (52.2) |  | 0.123 |
| No | 830 | (48.8) |  | 174 | (52.6) |  | 656 | (47.8) |  |  |
|  |  |  |  |  |  |  |  |  |  |  |
| *The analytical sample was derived from the enrollment data of an ongoing cluster randomized controlled trial, aimed at evaluating the effect of data-driven continuous quality improvement on long-term ART outcomes in Kinshasa, Democratic Republic of Congo. We retained participants that had available data on HBsAg testing. †Frequencies might not add up to n, because of missing data. ‡ Column percentage. §Facility at which participant was enrolled/tested. ¶Calculated using principal component analysis and categorized in three groups: the lower first two quintiles, the middle quintiles, and the last two quintiles. #Self-report of emotional or physical or sexual partner violence. **Self-report of disclosure of HIV status to anyone. Abbreviations: No, Number; VL, Viral load; SES, Socio-economic status; RNA, Ribonucleic acid, ART, Antiretroviral therapy. | | | | | | | | | | |
